# Mass Spectrometry-Based Quantification in Snake Venomics and Antivenomics: Current Trends and Future Perspectives

**DOI:** 10.3390/life16101636

**Published:** 2026-09-30

**Authors:** Nisha Reghu, Bipin Gopalakrishnan Nair, Muralidharan Vanuopadath

**Affiliations:** School of Biotechnology, Amrita Vishwa Vidyapeetham, Kollam 690 525, Kerala, India

**Keywords:** snake venom, mass spectrometry, proteomics, venomics, antivenomics, LC-MS/MS, quantification

## Abstract

Snake venomics and antivenomics approaches have increasingly relied on mass spectrometry to characterize the venom components and to evaluate the antivenom neutralization potential. However, accurate quantification remains essential for distinguishing biologically relevant toxins from snake venom and for translating proteomic data into meaningful clinical interpretations. This review article examines the role of quantitative analysis in snake venomics and antivenomics, with special emphasis on massspectrometry-based strategies used to estimate toxin abundance. This review summarizes the main applications and limitations of mass spectrometry in venom research covering bottom-up and top-down proteomics, venom databases, toxin quantification, and how MS-based quantification determines the efficacy of antivenom through antivenomics approaches. This review also explains why abundance alone cannot determine the biological or therapeutic importance of a toxin. The article discusses label and label-free based quantification, absolute quantification approaches, top-down venomics, mass spectrometry-based antivenomics, and the integration of proteo-transcriptomic workflows in snake venom research. These approaches can be affected by several factors including the problem of measuring toxin isoforms that share similar protein structures and toxin components that are important for antivenom recognition within complex venom samples. A conceptual overview is provided of how inaccurate quantification can influence venom proteome comparisons among similar and different species, as well as the interpretation of antivenom binding and neutralization potential. Reliable mass-spectrometry-based quantification will be critical for linking the venom composition with toxicity, antivenom performance evaluations in preclinical settings, and evidence-based strategies for snake venom research and snakebite management.

## 1. Introduction

Mass spectrometry-based snake venomics has enabled the comprehensive identification of venom toxins from medically important snake species. Even though the advent of advanced analytical techniques has accelerated venomics pipelines [1,2,3], strategies to accurately quantify venom toxins remains challenging. This is important because the biological and clinical significance of a venom depends not only on which toxins are present, but also on how much of each toxin is present. Though researchers have used mass spectrometry (MS)-based quantification strategies for estimating the relative abundance of snake venom toxins, it still will not be correlated with the actual abundance of individual proteins present in venom. This is mainly due to the limited information of snake venom protein sequences in the public database repositories, which results in over-representation of the actual venom proteome. Conventional venom proteomics generally rely on sequence similarity-based homology-driven approaches to identify venom toxins in crude venom. In most workflows, toxin identification depends mainly on the sample quality/protein abundance, instrument setting, tandem mass spectrometry data, often supported by peptide mass information, database matching, database quality, and search parameters. The identification of these protein sequences also relies on the sample preparation methods, including the proteases used for protein digestion (e.g., Trypsin, chymotrypsin, and V8 proteases), mode of digestion (in-gel or in-solution digestion), peptide pre-fractionation methods (HPLC, SDS-PAGE), type of mass spectrometer used for data collection, and data analysis workflows [4]. As a result, the same venom may yield different quantitative outputs depending on the workflow used.

Irrespective of these, some studies have also shown that de novo sequencing strategies combined with conventional proteomic workflows enable a better representation of the venom proteome [5,6,7]. However, none of these can be used to represent the actual venom proteome, quantitatively, and how confidently MS-derived abundance estimates reflect the absolute or relative abundance of individual toxins. Accordingly, the main issue is not whether mass spectrometry can be used in snake venom research, but how quantitative outputs should be interpreted in biologically and translationally meaningful ways. This review article therefore offers a comprehensive and integrative perspective on mass spectrometry-based quantification in snake venomics and antivenomics. It also examines how these measurements should be interpreted in biological and therapeutic contexts, an area that has not yet been systematically synthesized. Earlier reviews have covered the development of snake venomics, antivenomics, and the major proteomic platforms used to characterize venom components [8,9,10]. Calvete et al. reviewed the methodological basis of quantitative mass spectrometry in snake venom proteomics, including label-free and label-based methods, elemental MS, and the effects of peptide detectability, database quality, and protein inference on quantitative accuracy [11]. The present review extends this discussion by considering how quantitative results from venomics and antivenomics should be interpreted at successive molecular and biological levels. It brings together recent developments in top-down venomics, proteo-transcriptomics, targeted and elemental MS, and antivenomics within a single framework. The proposed pathway links toxin identification and abundance measurements with isoform or proteoform resolution, functional activity, antivenom recognition, neutralization, and therapeutic interpretation. It also emphasizes that toxin abundance does not establish toxicological activity, transcript abundance does not directly reflect the concentration of mature venom proteins, and antibody recognition does not demonstrate neutralization. The contribution of this review therefore lies in providing an interpretive and translational framework for quantitative venomics and antivenomics, rather than another summary of mass spectrometric platforms.

## 2. Role of Quantification in Snake Venomics and Antivenomics

In snake venom research, quantification is a scientific concern of utmost importance. It is currently shaping how we interpret venom variability, how we prioritize medically relevant toxins, and how we assess antivenom potency [10,12]. A simple toxin inventory is no longer enough, because the biological and clinical significance of a venom not only depends on which proteins are present, but also on their relative or absolute abundance, their functional potency, and their recognition by the antivenom [8,12]. Early studies based on gel electrophoresis combined with peptide mass fingerprinting helped to identify the venom proteins and assign them to various families. But they provided limited information about the precise abundance of individual components. Modern quantitative MS approaches can estimate relative or absolute abundance and therefore support comparisons among venoms from different species, populations, geographic regions, and life stages [10,11]. These measurements are useful for identifying abundant components and prioritizing toxins for further study, but abundance alone does not establish toxicological activity or clinical importance.

The level of molecular resolution is critical for biological interpretation. A family level measurement such as the abundance of phospholipase A_2_ (PLA_2_), snake venom metalloprotease (SVMP), snake venom serine protease (SVSP), or three-finger toxin proteins may include several closely related isoforms with different sequences and pharmacological properties. Shared peptides may further prevent reliable assignment to individual isoforms. An isoform- or proteoform-level interpretation requires unique peptides, intact mass measurements combined with sequence information, top-down analysis, or other orthogonal evidence [9,13]. Even when a particular isoform has been identified and quantified, functional assays are still required to determine its enzymatic, receptor binding, cellular, physiological, or in vivo activity.

Quantification is also relevant to antivenom evaluations, but its role must be distinguished from antibody binding and neutralization. In antivenomics, immunodepletion and immunoaffinity chromatography separate venom components according to their interactions with antivenom antibodies. Reversed-phased high-performance liquid chromatography (RP-HPLC) provides chromatographic fractionation, while MS identifies, validates, and estimates the abundance of toxins in the resulting fractions. First-, second-, and third-generation antivenomics have progressively improved the toxin-resolved assessment of antibody recognition and binding capacity (Table 1) [14,15,16]. However, antivenomics measures immunorecognition rather than functional neutralization. Neutralization must be established using biochemical, cellular, ex vivo, in vivo, or other validated functional assays.

Regional and interspecies differences in venom composition may help explain variations in antivenom recognition and clinical presentation. The regional venom variation of *Daboia russelii* and *Naja naja* shows differences in toxin levels and varied immunological cross-reactivity to the available polyvalent antivenoms, leading us to the fact of why quantitative analysis is necessary to understand the local patterns of envenoming [5,17]. Though these studies consistently show venom variation within the same species, published reports have not used the same methods for toxin quantification, which makes it difficult to compare the toxin levels between studies [5,17,18,19,20,21,22]. Therefore, these examples show that quantification is far more than technical details. It is central to understanding why venom variation matters medically and why antivenom evaluations cannot depend on qualitative observations alone. The study also highlights the need for more consistent quantitative workflows in snake venomics and antivenomics so that toxin abundance can be interpreted more confidently across studies. However, such relationships should be treated as hypotheses rather than direct causal conclusions. Comparisons among studies are also complicated by differences in venom sampling, pooling, chromatographic separation, MS acquisition, databases, quantification algorithms, and reporting practices [10,11]. Quantitative comparisons are therefore most reliable when the same analytical workflow, reference database, normalization procedure, and biological replication strategy are used. Besides just listing the toxins in a venom, quantification gives a clear picture of the toxins that are truly dominant and that are present in lower amounts but still show higher potency and how the venom composition varies across different geographical locations and species [10,12].

The discovery of venom-based therapeutics may depend on the biological activity, structural features, and pharmacological relevance, even when accurate toxin quantification is unavailable. Studies are being carried out on toxins that act on blood coagulation pathways, ion channels and immunological responses. Few examples include the antiplatelet drug eptifibatide, which is derived from rattlesnake venom [23], and captopril, derived from *Bothrops jararaca*, the first angiotensin-converting enzyme inhibitor used for hypertension and heart failure [24]. These examples highlight the potential of venom components as therapeutic leads and standard tools to investigate biologically relevant targets. The therapeutic value of a toxin depends on how strongly and selectively it is involved in inducing different pharmacological mechanisms and not only on its abundance. For example, PLA_2_s and SVMPs are the major immunomodulatory toxins that can often influence blood vessels, inflammation, immune cells, and tissue damage. However, individual toxin isoforms and less-abundant toxins may also have useful immunomodulatory effects [25]. In addition, the toxin abundance does not always reflect functional activity because some venom proteins appear as multimeric complexes or as subunits whose activity depends on their association with their partner proteins. Well-studied examples include crotoxin, a heterodimeric complex of crotapotin and a PLA_2_ subunit, β-bungarotoxins, and several C-type lectin-like proteins that function as oligomers or dimers [26,27]. Therefore, toxin abundance should support candidate selection but should not be the only factor used to judge therapeutic potential. In conventional bottom-up venomics, venom proteins are denatured, reduced and alkylated, and digested before LC-MS/MS analysis, so these native complexes are not measured as intact units. Instead, peptides from each component are quantified separately. As a result, the native stoichiometry is lost and the proteins may differ in abundance due to many factors such as the digestion efficiency, peptide recovery during sample preparation, LC separation, database matching, and shared peptides between homologous toxins [28]. When this happens, incorrect conclusions about which toxins drive coagulopathy, inflammation, vascular permeability, or tissue damage may be drawn. However, this has direct translational consequences, where the toxins selected for biomarker discovery, antivenom, or inhibitor development may be misprioritized, while the low-abundant, yet highly potent toxins are overlooked. In this way, inaccurate quantification does not give us wrong information about the protein identity; instead, it can influence how the pharmacological mechanisms are evaluated. For example, a recent study on opharin, a CRiSP from king cobra venom, demonstrated the enhanced release of inflammatory cytokines in macrophages and increased vascular permeability in vivo, even though it is only one component of a much more complex venom mixture [29]. This supports the broader view from reviews on venom-derived immunomodulators, which show that some venom toxins can affect immune cell numbers, migration, phenotypes, and cytokine release, while others may have anti-inflammatory effects. Together, these examples show that a toxin does not need to be one of the most abundant venom components to have a biological or therapeutic effect.

Accurate quantification is also important in the development of various adjunct therapeutics that are used as repurposed drug molecules complementing the antivenom. Small molecule inhibitors such as varespladib for PLA_2_, marimastat for SVMPs, and Phenylmethylsulfonyl fluoride (PMSF) for SVSPs have shown promising effects against different venoms [30]. But their effectiveness depends on the venom composition and also whether the toxin isoforms are susceptible to inhibition. Inaccurate quantification does not depend on the inhibitor specificity, rather it can affect which toxin families are prioritized as therapeutic targets. If the key toxins such as PLA_2_ or SVMPs are underestimated, the specific inhibitor for them such as varespladib or marimastat may also be overlooked. Therefore, accurate quantification is necessary to guide the selection of appropriate inhibitor combinations and to support the development of adjunct therapeutics [30]. Accurate quantification helps understand the venom composition in a better way and compare the venoms from different snakes or locations. This helps the researchers to understand which toxin families should be focused on for a detailed functional study. This has been shown in *Naja naja* and *Daboia russelii*, where the difference in toxin levels is associated with the clinical manifestations and how the antivenom works [5,31]. More broadly, venomics studies have also shown that quantitative profiles are useful for identifying which toxin families deserve closer mechanistic investigation [8]. The impact of inaccurate quantification is summarized in Figure 1, which discusses the analytical limitations followed by the translational consequences and the impact on patient care. Altogether, these examples show that quantitative venom proteomics is not just an analytical task. Measuring toxin abundance accurately is essential if venomics have to be translated into clinical and pharmacological use, whether for understanding venom action, evaluating antivenom performance, or identifying promising therapeutic leads.

## 3. Strategies Used in Mass Spectrometry-Based Quantification in Snake Venomics and Antivenomics

### 3.1. Label-Based and Label-Free Quantification of Snake Venom Toxins

Label-based and Label-free quantification in snake venom proteomics mostly relies on MS-based approaches. In snake venom proteomics, relative abundance is commonly estimated using shotgun LC-MS/MS workflows that apply spectral counting or ion-intensity-based approaches. Label-free proteomic approaches such as emPAI, NSAF, Top3, and iBAQ are commonly used for snake venom proteomics [11,32,33,34,35]. Besides label-free methods, labeling approaches such as tandem mass tags (TMTs) and iTRAQ (Isobaric Tags for Relative and Absolute Quantification) have also been used for comparative snake venom proteomics. Particularly, iTRAQ has been used for comparative snake venom proteomics, while TMTs have more recently been applied to quantify venom proteins in sea snake proteomes [36,37,38]. These techniques can be used for labeling the peptides from different snake venom samples and analyzing them together, which can be useful for comparing venom across species or different populations. A detailed comparison of different label-based and label-free quantification strategies is given in Table 2. iTRAQ-based proteomics have been recently used to compare the venom profiles of *Echis ocellatus*, *Naja nigricollis*, and *Bitis arietans* revealing the differences in the relative abundance of major toxin families [39]. TMT-based proteomics have also been used in combination with data-independent acquisition to characterize the venom proteome of sea snakes such as *Hydrophis curtus* providing information about PLA_2_s, three-finger toxins, and other venom components [40]. These examples show us how proteomics using labeling techniques can be used to compare multiple venom samples under controlled conditions, which can improve quantitative consistency by reducing between-run variation. However, such workflows need careful sample preparation and specialized data analysis. Their application in snake venomics is still hampered by the signal interference from the co-eluting peptides that can reduce the accuracy of the measured abundance. Other complications associated with isobaric labeling are the complexity of venom mixtures, the lack of suitable species-specific reference databases, increased analytical cost, and the absence of standardized workflows [41]. Thus, both label-based and label-free quantification remains the widely used strategy in quantitative snake venomics. However, most of these methodologies were originally developed for model organisms possessing well-annotated genomic or transcriptomic databases, which presents major limitations when applied to snake venomics, particularly for non-model species with incomplete sequence resources [11]. Although many studies have used this approach for snake venom proteome quantification, the resulting abundance estimates should be interpreted cautiously because they can be influenced by protein fractionation, enzymatic digestion, the type of mass spectrometer used for collecting the proteomics data, and the search engines and workflows used for data analysis. As a result, the same venom sample may yield somewhat different quantitative outputs depending on the analytical strategy used. At present, no universally standardized workflow is available for unbiased snake venom proteome quantification, which limits direct comparisons across studies.

Label-free methods estimate protein abundance from the MS1 signal intensity, extracted ion chromatograms, or computational metrices such as Top3, emPAI, iBAQ, and NSAF. Label-based methods use isobaric or stable isotope labeling including iTRAQ, TMT, SILAC, and ICAT. The table compares their analytical workflows, sensitivity, dynamic range, advantages, limitations, reproducibility, suitability for complex venoms, and application in snake venomics.

### 3.2. Absolute Quantification in Snake Venomics

Though relative quantification is widely followed in snake venomics, absolute quantification using mass spectrometry is attractive because it aims to measure the actual amount of each toxin in a venom sample rather than only its abundance relative to other components. This could improve comparisons between venoms from different populations or regions and provide a stronger basis for linking toxin levels with pharmacological effects, clinical manifestations and translational pharmacology [44,45]. However, its use in snake venomics is limited. There are reports that have used inductive coupled plasma mass spectrometry (ICP-MS)-based quantification strategies to quantify trace metals and other elemental constituents in whole snake venom. Sulfur-based LC-ICP-MS or ICP-QQQ-MS has been combined with molecular mass spectrometry to particularly quantify chromatographically separated sulfur-containing venom proteins. It measures the sulfur atoms present mainly in the cysteine and methionine residues, while molecular mass spectrometry is used in parallel to assign protein identities to the corresponding chromatographic peaks. When the identity and sulfur stoichiometry of a protein are known, the measured sulfur signal is converted to the absolute amount of that protein [44,46]. This approach is important since it quantifies intact venom proteins. However, the accurate quantification of individual venom toxins depends on efficient chromatographic recovery, correct protein identification, sequence information, accurate sulfur stoichiometry, and the adequate separation of closely related or co-eluting proteins. Sulfur measurement alone cannot identify the toxin responsible for a particular signal. The method is not universally applicable to proteins that lack sulfur-containing residues [44,46]. Another approach where absolute quantification is performed is ICP-MS, which measures the trace metals and other elements from crude snake venom. The resulting data describes the elemental composition of the venom but does not quantify intact toxin proteins or identify which proteins contain the measured element. Whole-venom elemental profiling may help to identify the metal co-factors and inorganic components that may or may not contribute to the overall toxicity of the venom [44,46]. However, digestion usually breaks down venom proteins and disrupts their original association with the metals. Through ICP-MS analysis, the absolute quantification of metal ions from several North American snake species has also been done [47]. However, the information obtained from this study is restricted only to the abundance of metal ions present in snake venom and not the venom proteome, so its value for interpreting toxin abundance and venom pharmacology is more limited. Further studies integrating whole-venom elemental profiling with molecular proteomics and functional assays are needed to determine whether variations in elemental composition are associated with toxin abundance, venom activity, and clinical manifestations of envenoming [43].

### 3.3. Top-Down Venomics

Top-down venomics is an emerging approach that may help overcome some of the limitations of bottom-up proteomic methods for toxin quantification. By analyzing intact toxins or proteoforms without prior digestion, top-down approaches can better preserve information on isoforms, post-translational modifications, and proteoform-level heterogeneity that may be obscured in peptide-based workflows. Native and top-down proteomics have been used to map proteoforms and protein complexes in king cobra venom, which is a good example of how top-down venomics can retain the information that is usually lost in bottom-up workflows, especially at the level of intact toxins and complexes [48]. Combined top-down and bottom-up approaches have also been used for *Echis carinatus sochureki*, which shows how top-down approaches can complement conventional proteomics by improving protein-level resolution in a medically important viper venom [49,50]. These may be especially relevant in snake venomics, where the value of top-down methods for protein-level resolution is highlighted. However, current limitations in sensitivity, proteoform resolution, throughput, and database support still restrict the routine application of top-down quantification to complex venom mixtures. For this reason, top-down venomics should currently be viewed as a promising complementary strategy rather than a full replacement for bottom-up quantitative workflows [13].

### 3.4. Proteo-Transcriptomic Approaches

Limited availability of protein information in the databases is considered one of the major problems associated with snake venom proteome quantification. Many snake species are poorly studied, and the available genomic, transcriptomic, and proteomic data are still found to be incomplete or are limited to a particular geographical region. If the toxins are missing in a sequence database, the MS spectra from them might get missed, or they may be get mismatched with other similar toxins. This can often give a false picture of which toxins are present and how abundant they are [11]. Proteo-transcriptomics helps address this problem by incorporating venom gland RNA-sequencing data into the proteomic workflow. This process includes quality assessment and the trimming of raw reads, de novo transcript assembly using tools such as Trinity, coding sequence and open reading frame prediction, the translation of transcripts into predicted protein sequences, toxin annotation, the removal of abundant sequences and contaminants, and the construction of a species-specific predicted proteome database. A conceptual framework based on this workflow has been given in Figure 2.

Venom gland transcriptomics and venom proteomics provide complementary information rather than interchangeable measurements. Transcriptomic analysis describes the repertoire and relative expression of toxin genes, whereas proteomics confirm which predicted proteins are present in the venom and provide their relative or absolute abundance. Modahl et al. emphasized that species-specific venom gland transcriptomes improve mass spectrometric identification by providing sequences that are often absent from public or taxonomically distant databases [50]. Comparative studies have also shown the value of analyzing both datasets in the same biological system. Hofman et al. combined venom gland transcriptomics with venom proteomics across four lineages of *Crotalus cerastes*. Their results showed limited overall differential expression despite individual variation and demonstrated that conclusions about venom evolution depend on the number of individuals sampled, the phylogenetic framework used, and the distinction between transcript expression and venom composition [51] Similar proteomic–transcriptomic approaches have been used to investigate toxin diversity, sequence variation, and the molecular evolution of venom components [1].

The integration of these datasets improves toxin identification, peptide assignment, sequence coverage, and isoform discrimination. It can also reveal cases in which highly expressed transcripts are poorly represented in the secreted venom or where abundant venom proteins are not proportionally reflected at the transcript level. Such discrepancies may result from translational regulation, proteolytic processing, secretion, storage, degradation, the sampling time, or analytical bias [52]. Transcriptomic abundance should therefore be treated as a measure of toxin-gene expression, whereas protein abundance must be determined from the venom proteomic data.

In quantitative venomics, the practical value of proteo-transcriptomics lies in its ability to support a species-specific MS search database. After transcriptome quality control, de novo assembly, coding sequence prediction, and the translation of toxin transcripts, the resulting predicted protein database can be used to search raw MS data from in-gel or in-solution venom digests. High-confidence unique peptides can support a protein- or isoform-level assignment, while shared peptides should be reported at the protein family or protein group level. The identified proteins can then be quantified using the MS1 intensity, extracted ion chromatograms, spectral counting, label-based ratios, targeted MS, or calibrated elemental MS (Figure 2). Proteo-transcriptomics therefore improve the reliability of protein identification and quantitative interpretation but does not convert transcript counts directly into mature toxin concentrations.

By integrating venom gland transcriptomic data with proteomic workflows, researchers can expand the searchable sequence space, improve peptide matching, and obtain more reliable toxin annotation, especially in species where public databases are sparse or taxonomically distant [53]. The importance of species-specific study is well illustrated by the Indian cobra reference genome and transcriptome study, which enabled the comprehensive identification of venom toxins and provided a much stronger framework for venom characterization [22]. This study is essentially relevant for quantification because it shows that improved genomic and transcriptomic databases not only increase toxin identification but also enhance the reliability of the venom composition and quantitative interpretation by providing more accurate protein annotation. However, transcript abundance should not be treated as a direct measure of the mature venom protein concentration. Differences in translation, proteolytic processing, secretion, storage, degradation, post-translational modification, and the sampling time can produce substantial transcript protein discordance. The transcriptome-derived database therefore supports protein identification and isoform assignment, whereas the abundance of mature venom proteins must be determined from the proteomic data. Although incomplete toxin databases have been discussed before [11,54], their importance is that missing or incorrect annotations can affect later quantitative analysis and the biological or clinical conclusions drawn from it.

### 3.5. Mass Spectrometry-Based Antivenomics

For checking the antivenom immunological cross-reactivity studies using snake venom, ELISA and Western blotting approaches have been widely used [55], but they are mainly qualitative and provide limited information about how much of each toxin is captured and how specific that recognition is. Therefore, they cannot accurately determine the relative amounts of recognized and non-recognized venom components. To determine and quantify the amount of antivenom-bound and antivenom-unbound snake venom proteins, MS-based antivenomic approaches have been widely used (Table 1) [56].

Antivenomics combine antibody-mediated separation with chromatographic and mass-spectrometric analyses. Through first-, second-, and third-generation antivenomic approaches, there has been improved quantitative study of antibody–toxin interactions and a better understanding of antivenom paraspecificity and cross-reactivity across different snake venoms [14,15,16]. First-generation antivenomics rely completely on the concept of immunoprecipitation-based immunodepletion, where the antivenom-recognized venom components are removed as precipitated antigen–antibody complexes. The remaining supernatant is analyzed via RP-HPLC and compared with a control venom aliquot. The integration of differences in corresponding chromatographic peak areas provides an estimate of the degree of immunodepletion and, consequently, the recognition of individual venom components by the antivenom. But this was limited by its indirect readout and relatively poor quantification [11]. In second-generation antivenomics, immunoprecipitation was replaced by an immunoaffinity method. Antivenom is covalently coupled to an affinity matrix to separate venom components into bound and unbound fractions. The retained toxins are subsequently released by modifying the pH. The recovered fractions along with naive IgG are analyzed via RP-HPLC to quantify the degree of immunorecognition of individual venom components [15]. Second-generation antivenomics have been widely applied to evaluate the homologous and paraspecific coverage of antivenoms such as EchiTAb-Plus-ICP against Echis, Bitis, and African spitting cobra venoms. It showed broad cross-reactivity, but also identified important gaps in recognition, particularly among low-molecular-mass proteins with poor immunogenicity [15]. Third-generation antivenomics mark a significant step forward in the preclinical assessment of antivenoms [16]. This enhances traditional in vivo neutralization assays by offering detailed qualitative and quantitative information on antibody–toxin interactions. By employing immunoaffinity columns loaded with increasing amounts of venom, this method determines the maximum binding capacity of antivenom for specific venom components. It can also determine the proportion of venom-specific antibodies in an antivenom preparation. Size-exclusion HPLC analysis can measure antibody–toxin complexes that form when venom is incubated with antivenom [16]. Studies of antivenoms such as Bothrofav for *Bothrops lanceolatus* can quantify not only which toxins are recognized but also the proportion of therapeutic antibodies effectively engaged in toxin binding, allowing a more mechanistic interpretation of neutralization potencies. Thus, third-generation antivenomics provide a more quantitative assessment of antibody–toxin binding while retaining immunoaffinity chromatography as the separation principle [16].

Immunoprecipitation-based immunodepletion and immunoaffinity chromatography are antibody-mediated separation procedures. RP-HPLC provides chromatographic fractionation, whereas mass spectrometry provides identification, as well as quantitative information, when appropriately validated. Mass spectrometry does not itself separate toxins according to antibody recognition [15,16]. More recently. LC-MS-based in-solution approaches have been proposed as a possible further development or as a potential fourth-generation antivenomic framework [57]. In contrast to the earlier immunoprecipitation- and immunoaffinity-based workflows, these methods analyze venom–antivenom mixtures directly after incubation in solution using reverse-phase LC-MS. Untreated venom is first analyzed via LC-MS to establish the signal of each intact protein. The venom is then incubated with antivenom and is analyzed again. A change in the toxin’s chromatographic peak or MS1 signal may indicate antibody binding. This comparison can show which venom components are targeted by the antivenom [57]. If the analytical resolution is sufficient, individual toxin isoforms may also be distinguished. However, these results indicate antibody recognition and do not directly prove toxin neutralization.

Antivenomics measure how well the toxins are recognized by the antibodies rather than whether those toxins are neutralized. The presence of a toxin in either the antibody-bound or the immunodepleted fraction indicates that an immune reaction has occurred under the test conditions, but it does not prove that the toxin’s harmful effects are blocked. Antivenomic data should therefore be considered alongside biochemical and cell-based assays, in vitro neutralization assays, and, when relevant, preclinical efficacy studies [58]. The results can also be affected by several experimental factors that include the venom-to-antivenom ratio, immunoaffinity column capacity, antibody saturation, nonspecific binding, and differences in the analytical workflow, highlighting the need for improved methodological standardization across studies [16].

## 4. Conclusions and Future Perspectives

As directed by the World Health Organization, one of the major goals is to reduce snakebite deaths to half by the year 2030 [59]. Several national and international initiatives have been introduced to improve the treatment and prevention of snakebite envenoming and are put in place to streamline effective treatment and preventive measures [60,61,62,63]. Achieving this goal will require not only better antivenoms but also a more accurate understanding of the venom composition and biological activity [64,65].

Future research should focus on three priorities: standardized methods, improved toxin identification, and functional antivenom validation. Quantitative venom studies should clearly report the venom origin, pooling, storage, sample preparation, MS parameters, database construction, normalization, and biological replication. Toxin identification should be improved through high-resolution LC-MS/MS, species-specific transcriptome databases, and de novo peptide sequencing, especially for poorly studied species and toxins [50,52]. Proteo-transcriptomics should form an important part of this framework. Venom gland transcriptomics can provide species-specific toxin sequences and transcript-level information, whereas proteomic analysis confirms which predicted proteins are present in crude venom. Integrating these approaches can improve peptide assignment, sequence coverage, isoform discrimination, and protein inference, particularly for medically important species with limited reference data. However, transcript abundance should not be treated as a direct measure of venom protein abundance; reliable protein quantification requires calibrated proteomic methods [52,53]. Shared resources such as VenomsBase may further improve data comparisons and toxin annotation [66].

Finally, proteomic findings must be linked to biological activity and antivenom performance. Toxin abundance cannot establish toxicity, and antibody binding does not necessarily indicate neutralization or clinical protection. Functional assays, antivenomics, and appropriate preclinical neutralization studies should therefore be integrated with quantitative MS and proteo-transcriptomic data. This approach will improve the interpretation of venom complexity and support the development of more effective antivenoms.

## Figures and Tables

**Figure 1:**
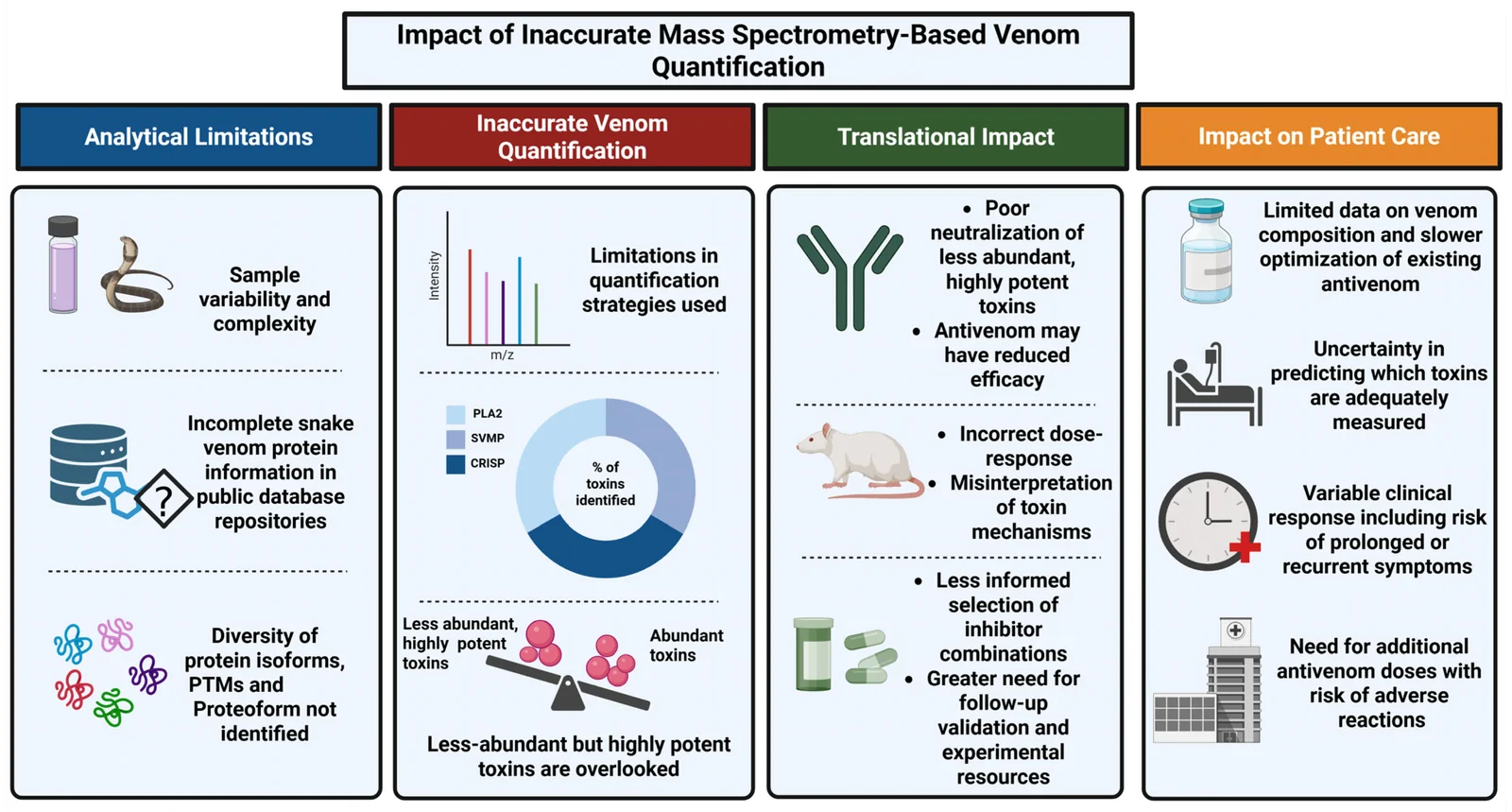
Conceptual overview of the impact of inaccurate mass spectrometry-based venom quantification (Created in BioRender. Nair, B. (2026) https://BioRender.com/fwkvi1l, accessed on 13 August 2026).

**Figure 2:**
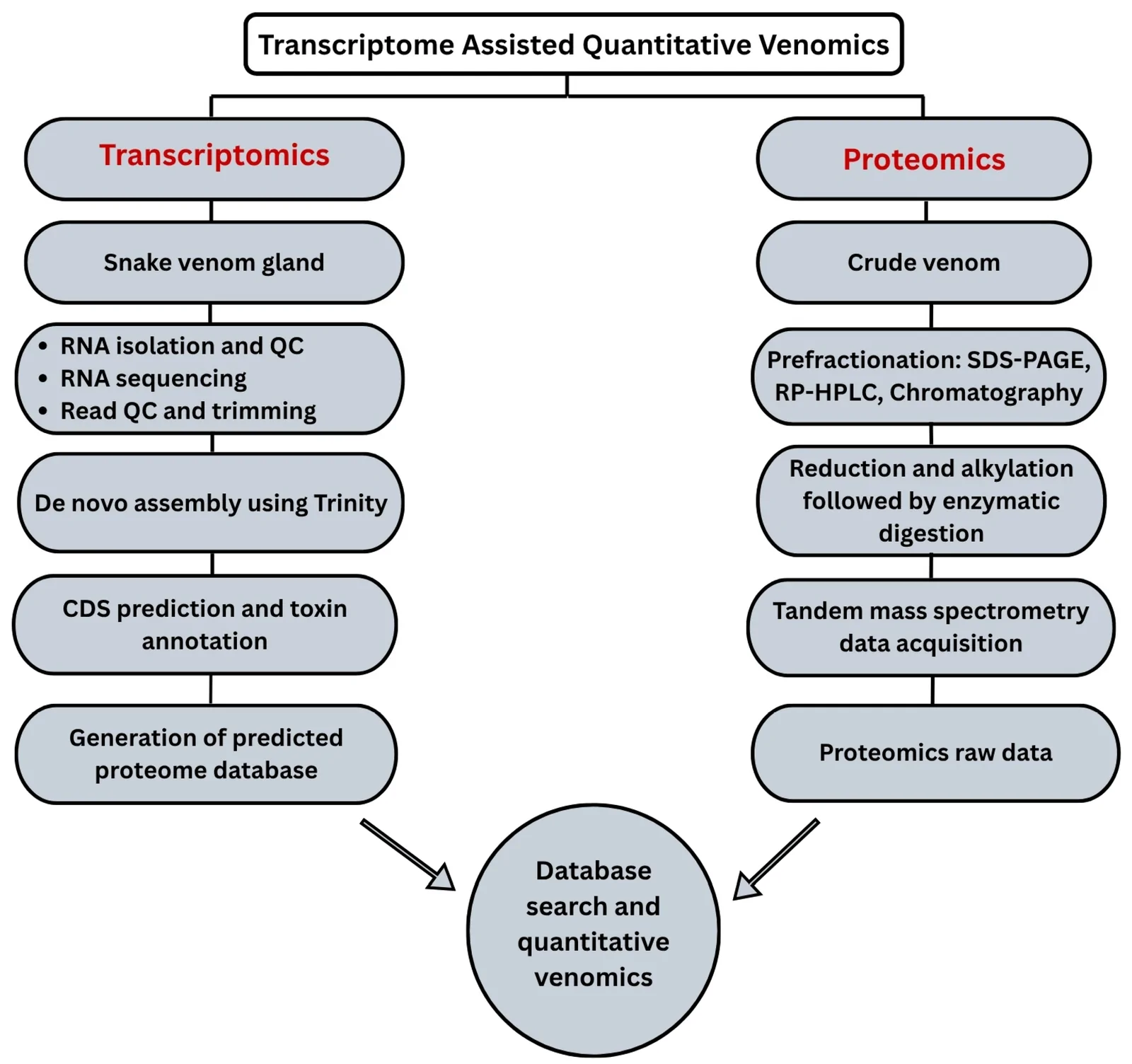
A conceptual framework for transcriptome-assisted quantitative snake venomics.

**Table 1:** Evolution of antivenomics strategies through different generations, highlighting their analytical technologies, advantages, and limitations.

Sl No	Antivenomics Strategy	Technologies Employed	Advantages	Limitations	Reference
1.	First-generation antivenomics	Immunodepletion followed by RP-HPLC	Enables characterization of venom components that are recognized, not recognized, and immunodepleted by the antivenom	Mainly limited to whole IgG antivenoms, Fails to detect or quantify low-abundance toxins	[14]
2.	Second-generation antivenomics	Uses immunoaffinity-based matrix capture to isolate bound vs. unbound venom fractions	Relative quantification, compatible with F(ab)_2_ antivenoms, the columns can be reused, improving applicability, reducing assay cost, and enhancing reproducibility	Affected by incomplete immunodepletion, non-specific binding, and differences in toxin abundances	[15]
3.	Third-generation antivenomics	Immunoaffinity chromatography with LC-MS/MS	Determines maximum binding capacity of antivenom and estimates toxin specific antibody fractions, quantitatively	This is also an in vitro binding assay; binding does not equal neutralization	[16]

**Table 2:** Comparison of label-based and label-free mass spectrometry-based quantification strategies.

**Label-Based Quantification Strategies**							
Quantification strategy	Analytical workflow	Sensitivity and dynamic range	Advantages	Limitations	Reproducibility	Suitability for venom complexity	Application in snake venomics
TMT [36]	Peptides are labeled with tandem mass tags, combined, and analyzed via LC-MS/MS.	Allows simultaneous analysis of multiple samples and detects low-abundance peptides	Allows simultaneous comparison of several samples in one MS experiment	Signal interference from co-isolated peptides can reduce quantitative accuracy, and the labeling reagents are relatively expensive	Good reproducibility with fractionation and quality controls	Suitable for comparing complex venoms, although pre-fractionation may be needed to improve peptide identification and quantitative accuracy	Suitable for comparative venom analysis, though its application is less documented than iTRAQ
iTRAQ [37]	Peptides from different venom samples are labeled with isobaric tags, pooled, and analyzed via LC-MS/MS.	Offers multiplexed sensitivity down to femtomole range	Allows simultaneous comparison of several samples in one MS experiment	Ratio compression, reporter-ion interference, incomplete labeling, and co-isolation can reduce quantitative accuracy	Good reproducibility with pooled quality controls, normalization, and batch correction	Useful for comparing complex venoms, although spectral interference increases with sample complexity	Applied to comparative snake-venom proteomics, including ontogenetic and interspecies comparisons
SILAC [42]	Venom-producing cells or experimental systems are supplied with light or heavy amino acids. Labeled and unlabeled proteins are mixed, digested, and analyzed via LC–MS/MS.	Provides more accurate dynamic range compared to iTRAQ, since it measures differences at MS1 level rather than using reporter ions in MS2	Labeling occurs before sample preparation and can reduce technical variation	Limited applicability to snake venom, requires controlled biological systems	High reproducibility for controlled biological systems; incomplete isotope incorporation and differences in cell growth can introduce variability	Limited suitability for routine venom comparison	Limited direct use in snake venomics
ICAT [43]	Cysteine residues are labeled with light or heavy isotope, and labeled peptides are then enriched through affinity capture and analyzed via LC-MS/MS.	Good sensitivity for cystine-containing peptides	Improves detection of low-abundance cysteine-containing peptides	Does not quantify proteins lacking suitable cysteine residues	Moderate to good when labeling, enrichment, and digestion are carefully controlled	Applicable to complex venoms, but may not quantify all toxin families and isoforms	Potentially applicable but not widely reported
**Label-Free Quantification Strategies**							
Quantification strategy	Analytical workflow	Sensitivity and dynamic range	Advantages	Limitations	Reproducibility	Suitability for venom complexity	Application in snake venomics
NSAF [33]	Spectral counts are divided by protein length and normalized to the sum of length-corrected spectral counts across the sample.	Provides moderate sensitivity for abundant proteins. Low spectral counts are unstable, and spectral-count saturation limits the effective dynamic range	Simple, inexpensive, and corrects partly for the tendency of longer proteins to generate more spectra	It is affected by peptide detectability, protein length, shared peptides, and spectral-count saturation	Moderate to good for abundant proteins when replicate number and data processing are standardized	Useful for comparing major toxin families, but less reliable for rare toxins and closely related isoforms	Useful for comparing toxin abundance in shotgun venom proteomics when MS/MS spectral counts are available
iBAQ [34]	The intensities of all identified peptides assigned to a protein are summed and divided by the number of theoretically observable peptides. Relative iBAQ can be obtained by normalizing each value to the total iBAQ signal.	Uses more peptide signals than Top3, but low-abundance proteins may be affected by missing data and ionization differences	It adjusts for protein length to show the relative abundance and ranks different proteins across samples	Results are affected by shared peptides, incomplete databases, missed cleavage, and incorrect protein inference	Moderate to good with consistent LC–MS acquisition, normalization, and adequate replication	Useful for comparative profiling of complex venoms when species-specific databases are available	Potentially useful for comparative venom-proteome profiling
emPAI [32]	LC-MS/MS spectral counting is used. The number of observed peptides is divided by the number of theoretically observable peptides.	Useful mainly for medium- and high-abundance proteins	Easy to calculate from existing shotgun proteomic data. It does not require isotopic labeling or protein-specific standards	Affected by digestion, peptide detectability, shared peptides, database quality, and MS sampling	Moderate. Reproducibility improves with biological and technical replicates and consistent data acquisition	Good for comparing major protein families, but less reliable for low-abundance proteins and closely related isoforms	Provides approximate protein abundance and is considered semi-quantitative
Top3 [35]	Bottom-up LC-MS or LC-MS/MS is used to identify peptides. The average intensity of the three most intense quantifiable peptides assigned to a protein is used to estimate abundance.	Most reliable for abundant proteins with at least three well-detected peptides	Easy to use and is less biased by protein size; can compare protein abundance between samples	Results depend on peptide selection, ionization efficiency, digestion, shared peptides, and missing values. It may not distinguish closely related isoforms	Moderate to good when LC-MS conditions, peptide selection, and normalization are consistent	Suitable for moderately complex venoms, especially after chromatographic fractionation. Less suitable for minor toxins and proteins with limited peptide coverage	Useful for comparing the relative abundance of identified venom proteins, but it is not truly quantitative without standards

## Data Availability

No new data were created or analyzed in this study. Data sharing is not applicable to this article.
